# Antepartum Rupture of the Posterior Uterine Wall in a Woman With Two Previous Cesarean Deliveries

**DOI:** 10.7759/cureus.52517

**Published:** 2024-01-18

**Authors:** Marina Gato, Catarina Castro, Luísa Pinto

**Affiliations:** 1 Department of Obstetrics, Gynecology and Reproductive Medicine, Centro Hospitalar Universitário Lisboa Norte, Lisboa, PRT

**Keywords:** neonatal outcomes, hysterectomy, cesarean section, uterine rupture, pregnancy

## Abstract

Uterine rupture is a rare pregnancy complication. In patients with a previous cesarean delivery, it usually involves the scarred area. Uterine rupture of the posterior wall is even rarer and mostly described during labor. Conditions that confer fragility to the posterior uterine wall have been associated with an increased risk of uterine rupture. There are very few cases of spontaneous posterior uterine wall rupture in a non-labor setting in pregnant women without risk factors.

We report the case of a pregnant woman admitted to the hospital due to placental abruption at 26 weeks’ gestation. Once fetal and maternal stability were assured, expectant management was maintained. At 29 weeks, an emergent cesarean delivery due to fetal bradycardia was performed, and a large rupture of the posterior uterine wall was diagnosed. Subsequently, a hysterectomy was performed. The patient was discharged nine days after the procedure and the newborn on the 64th day of life.

## Introduction

Uterine rupture is a rare pregnancy complication, occurring in approximately 5.3/10,000 pregnancies. This rate increases to 94/10,000 in pregnant women with a scarred uterus [1]. Generally, the uterine rupture involves the scarred area, specifically the lower segment of the anterior wall, where the previous hysterotomy was performed. Uterine rupture of the posterior wall is even rarer and mostly described during labor or induction [2-4]. We present the case of a posterior uterine wall rupture in the early third trimester in a non-labor setting.

## Case presentation

A pregnant woman, gravida 3 para 2, presented to our emergency room at 26 weeks` gestation due to severe vaginal bleeding. She had two previous cesarean deliveries with low transverse incisions, both due to HELLP (Hemolysis, Elevated Liver enzymes, Low Platelets) syndrome. At admission, ultrasound was performed, which revealed normal fetal heart rate, a posterior placenta with a 60x49mm detachment, a 100x80mm subchorionic hematoma in the anterior wall of the uterus, and a closed cervix. She was admitted to the labor ward, and betamethasone and magnesium sulfate were administered. Since vaginal bleeding was mild and hemoglobin values were stable at 10.2 g/dL, she was transferred to the maternal-fetal ward for expectant management. Weekly obstetric ultrasound scans were performed revealing minor enlargement of the hematomas: retroplacental hematoma measuring 67x72mm and subchorionic hematoma measuring 130x69mm. Maternal and fetal conditions remained stable.

**Figure 1:**
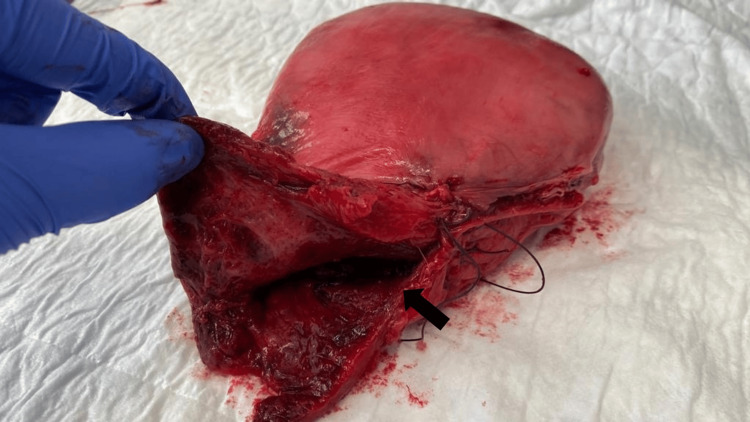
Photo of the removed uterus. The rupture, indicated with an arrow, extends from the middle of the uterine body until the cervix and upper third of the vagina.

At 29 weeks` gestation, the patient complained of acute severe abdominal pain and an episode of severe vaginal bleeding with spontaneous resolution. Because of fetal bradycardia, an emergent cesarean delivery was performed. Fetal extraction was challenging, and there was a need for vertical extension of the transverse uterine incision. A newborn weighing 1,146 g, with Apgar scores of 7 and 8 at 1 and 5 minutes, respectively, was delivered. The amniotic fluid was bloody and contained multiple clots. After hysterorrhaphy, a longitudinal defect in the midline of the posterior uterine wall, extending from the uterus to the upper third of the vagina, was observed (Figure 1). Because of the huge extension of the ruptured area, a hysterectomy was performed, and although uterine anatomy was distorted, the procedure was uneventful, and the ovaries were preserved. Intraoperatively, transfusion of 2 packs of red blood cells was necessary.

The postoperative period was uneventful, with no need for intensive care unit admission, and the patient was discharged nine days after the surgery, with a hemoglobin level of 9.2 g/dL. The newborn was discharged on the 64th day of life after recovery in the neonatal intensive care unit. Along with complications of prematurity, subgaleal hematoma and hemorrhagic shock were also diagnosed.

Histopathologic examination confirmed rupture of the uterine wall and the presence of a retroplacental hematoma, with no other anomalies of the placenta.

## Discussion

Uterine rupture is a rare but potentially catastrophic complication of pregnancy. The reported incidence of maternal mortality associated with uterine rupture is quite distinct, with the largest published case series from a Canadian study reporting one death in 500 cases [1]. The reported incidence of neonatal/perinatal mortality varies from 6% to 26% [2].

Uterine rupture after a previous cesarean delivery usually occurs at the site of the previous scar due to the inelastic nature of the scarred tissue [3]. Several studies, however, have reported that the rigid anterior lower segment may cause an abnormal distribution of forces, which may predispose to excessive stretching and thinning of the posterior wall, leading to an atypical rupture of the healthy tissue [3-5]. This could explain the rupture of the posterior uterine wall during labor. Posterior uterine wall rupture in pregnant women in the first or second trimester or in the third trimester but not in labor is an even rarer phenomenon [6,7].

Clinical presentation of posterior uterine rupture is similar to that of anterior wall rupture. The most frequent sign is a pathological cardiotocogram with fetal bradycardia. Acute abdominal pain is also an important feature. Vaginal bleeding can be of variable intensity, depending on the extension of the rupture to the cervix or vagina and on the presence of rupture of membranes. If the hemorrhage is severe, maternal hemodynamic instability may be present. Finally, loss of fetal station may also be an important sign [2,8]. In pregnant women presenting with acute abdominal pain, vaginal bleeding, and fetal heart rate abnormalities, uterine rupture should always be considered.

A high suspicion of uterine rupture should prompt an emergent cesarean delivery, and after fetal extraction, a conservative approach with suture of the ruptured area should be attempted. However, depending on the length of the rupture, difficulty in hemostasis, and maternal hemodynamics, a hysterectomy may have to be considered. Hysterectomy is performed in 14 to 33% of the patients with uterine rupture [9]. In our case, the extent of the rupture was the main determining factor toward the decision to perform hysterectomy. Given the necessity of performing hysterectomy following an episode of hemorrhage, and according to the WHO criteria, we can define this as a maternal near-miss case [10].

Several factors seem to increase the risk of uterine rupture in women with a scarred uterus, such as previous uterine rupture, induction of labor (especially if prostaglandins are used), history of two or more cesarean deliveries, shortened interpregnancy interval, and no previous vaginal delivery [9,11-13]. Some authors have also reported some risk factors specific for posterior uterine wall rupture, namely situations or procedures that confer weakness to the uterine wall, such as previous curettage, previous myomectomy, congenital malformations of the uterus, and abnormal placentation [4,7,14-16].

Among the cases described in the literature, we mainly found ruptures of the posterior uterine wall in the third trimester of pregnancy, during labor induction, intrapartum, or in patients with at least one of the risk factors previously mentioned. There are very few cases of spontaneous posterior uterine rupture in non-labor settings in pregnant women without the risk factors listed above.

In this case, the patient had been admitted to the hospital with the diagnosis of placental abruption. Obstetric ultrasounds revealed the presence of two hematomas, one subchorionic in the anterior wall of the uterus and the other retroplacental. The placenta was posterior, and a mild enlargement of the hematoma was observed during the admission to the ward. We hypothesized that the uterine rupture could be related to the evolving hematoma expanding between the placenta and the uterine wall; however, no similar cases have been previously described in the literature.

## Conclusions

This report describes a case of posterior wall uterine rupture in a non-labor setting in a pregnant woman without known risk factors. Uterine rupture is a rare but potentially catastrophic complication of pregnancy. Posterior uterine wall rupture is even rarer, especially in non-labor settings. In pregnant women presenting with acute abdominal pain, vaginal bleeding, and fetal heart rate abnormalities, uterine rupture should always be considered. Depending on the length of the rupture, difficulty in hemostasis, and maternal hemodynamics, hysterectomy can be considered.
